# Community-based heat-sensitive moxibustion for primary hypertension: study protocol for a randomized controlled trial with patient-preference arms

**DOI:** 10.1186/s13063-022-06092-4

**Published:** 2022-02-16

**Authors:** Xu Zhou, Shuqing Li, Ling Li, Guihua Deng, Li Dai, Luyu Chai, Qingni Wu, Ziqian Yao, Minchao Deng, Weifeng Zhu, Yong Fu, Xin Sun

**Affiliations:** 1 Evidence-based Medicine Research Center, Jiangxi University of Chinese Medicine, Nanchang City, Jiangxi China; 2 Chinese Evidence-based Medicine Center, West China Hospital, Sichuan University, Guo Xue Xiang No.37, Chengdu City, 610041 Sichuan China; 3 First Department of Acupuncture and Moxibustion, The Affiliated Hospital of Jiangxi University of Chinese Medicine, Bayi Avenue No.445, Nanchang City, 330006 Jiangxi China

**Keywords:** Heat-sensitive moxibustion, Primary hypertension, Community healthcare, Pragmatic trial

## Abstract

**Background:**

Low- and middle-income countries have a high prevalence of primary hypertension, but its treatment and control are often low. Heat-sensitive moxibustion (HSM), an innovative acupoint stimulation technique, may be effective for treating hypertension and thus used appropriately in primary healthcare. The objective of this study is to investigate whether HSM is effective and safe for the treatment of primary hypertension in the community.

**Methods:**

This study is a multicenter, pragmatic, randomized controlled trial (RCT) with patient-preference arms. Four hundred patients with primary hypertension from seven communities will be enrolled. Initially, the communities will be randomly assigned into two study clusters, one using compulsory randomization and the other allowing treatment selection by patient preferences. Then, patients in the compulsory randomization cluster will be randomized to receive HSM plus their original antihypertensive regimen (HSM group) or only their original antihypertensive regimen (control group) for 6 months. Patients in the patient preference cluster may choose to receive HSM or control if they have a preference; otherwise, patients will be randomly assigned. The primary outcome is the change in systolic blood pressure from baseline; secondary outcomes include change in diastolic blood pressure, dosage of antihypertensive drugs, quality of life (QoL), severity of hypertensive symptoms, and incidence of cardiovascular events. Patient compliance with the HSM regimen, the cost-effectiveness ratio, and safety outcomes will also be evaluated. Outcome data will be collected at 6 monthly visits.

**Discussion:**

This trial will provide important evidence regarding HSM as a technique for primary hypertension in primary healthcare settings. Given the randomization with patient preferences considered, the trial will also allow analyzing patient-preference effects and the comparison of randomized and nonrandomized samples, to improve the robustness and extrapolation of study conclusions.

**Trial registration:**

ClinicalTrials.govNCT04788563. Registered on March 9, 2021.

**Supplementary Information:**

The online version contains supplementary material available at 10.1186/s13063-022-06092-4.

## Background

Hypertension is a major risk factor for cardiovascular disease and premature death and affects approximately one-third of the population worldwide [1]. Low- and middle-income countries have a higher burden of hypertension, resulting in fewer disability-adjusted life years and higher hypertension-related mortality than high-income countries, highlighting the challenge of hypertension management in these countries [1, 2]. In China, for example, one episode of intensive blood pressure control costs up to $766, which in 2015 was equivalent to 22.7% of the average citizen’s annual disposable income [3], while the rate of optimally controlled hypertension has remained low in the past dozen years [4].

A key reason for the imbalance in cost-effectiveness in low- and middle-income countries may be that primary healthcare interventions for hypertension are not yet well developed [5, 6]. Despite consensus on the effects of reducing blood pressure and cardiovascular disease risk with long-term adherence to antihypertensive drugs, under a single hospital management model, a large proportion of patients cannot maintain their blood pressure at the ideal level [7]. Indeed, patients often discontinue their medication because of inconvenience in accessing medical care or concerns about adverse effects. Therefore, developing appropriate treatment regimens for hypertension that are effective, safe, and inexpensive and can be widely popularized in the community is warranted to compensate for the inadequacy of the hospital management model [8].

Moxibustion is an acupoint stimulation therapy that has been employed as a complementary and alternative therapy in Chinese medicine for thousands of years. In moxibustion, burning moxa sticks are used to stimulate specific acupoints with heat. Specifically, when patients receive moxibustion therapy, they may be stimulated with “heat-sensitized sensations”, as follows: heat penetration, in which the patient can feel the moxa heat penetrating through the skin into deep tissues (the skin surface may or may not be hot); heat diffusion, in which the patient can feel the moxa heat spreading to the surrounding area (the vicinity of the acupoint may or may not be hot); heat conduction, in which the patient can feel the moxa heat transferring a distance in one direction; and nonthermal sensation, in the patient they may feel a creeping sensation, swelling sensation, soreness, coolness, etc. [9]. When patients experience these special sensations, the efficacy of moxibustion can be significantly enhanced compared with conventional moxibustion for multiple diseases, such as osteoarthritis [10], lumbar disc herniation [11], and bronchial asthma [12]. A moxibustion technique with the purpose of stimulating heat-sensitized sensations and applying a saturating moxibustion dose until the sensations disappear is called heat-sensitive moxibustion (HSM).

Overall, HSM has the potential to become an appropriate technique for the management of hypertension in the community because it is simple, convenient, effective, and inexpensive. For example, moxa heat stimulation at acupoints has been demonstrated to induce positive changes in the levels of renin activity, atrial natriuretic peptide, and aldosterone in plasma, resulting in a reduction in blood pressure [13]. HSM is also considered to reduce blood pressure by modulating the expression of the mammalian target of rapamycin and decreasing the level of oxidative stress in the body [14]. A meta-analysis including 18 randomized controlled trials (RCTs) found that moxibustion reduced systolic blood pressure (SBP) by 7.85 mmHg and diastolic blood pressure (DBP) by 4.09 mmHg in patients with primary hypertension over a median 4-month course, although it should be noted that the evidence was not for HSM specifically and the quality of evidence was affected by the risk of bias [15]. The HSM technique is also easy to learn and perform; after receiving a short training session, patients can administer moxibustion at home, by themselves or with the help of family members. Moreover, self-administration of HSM becomes more convenient with the use of a moxibustion device (e.g., moxibustion jar).

Nevertheless, implementing HSM for the treatment of hypertension in the community still requires evidence regarding the following critical questions: Does RCT evidence support that HSM is effective for hypertension? Can HSM replace some doses of antihypertensive drugs? What are the safety, compliance, and cost-effectiveness of HSM for hypertension in the community setting? To answer these questions, we plan to perform a pragmatic RCT with patient preference consideration. We hypothesize that adding daily HSM self-management to traditional antihypertensive treatment/no treatment will result in better blood pressure control efficacy than the original antihypertensive regimen, with good safety and lower costs. Given the real-world situation that patients in community settings may adapt different antihypertensive regimens, this pragmatic trial will be based on an add-on comparison (i.e., HSM + original antihypertensive regimen vs. original antihypertensive regimen) to validate the hypotheses, which will also facilitate maintaining patients’ original preference in the patient-preference arms and observing changes in dosage of antihypertensive drugs over the study period.

## Methods/design

### Study design

The study protocol has been registered at ClinicalTrials.gov (Identifiers: NCT04788563), a registry including all items from the WHO Trial Registration Data Set. We developed this article following the Standard Protocol Items: Recommendations for Interventional Trials (SPIRIT) guidelines [16].

**Fig. 1:**
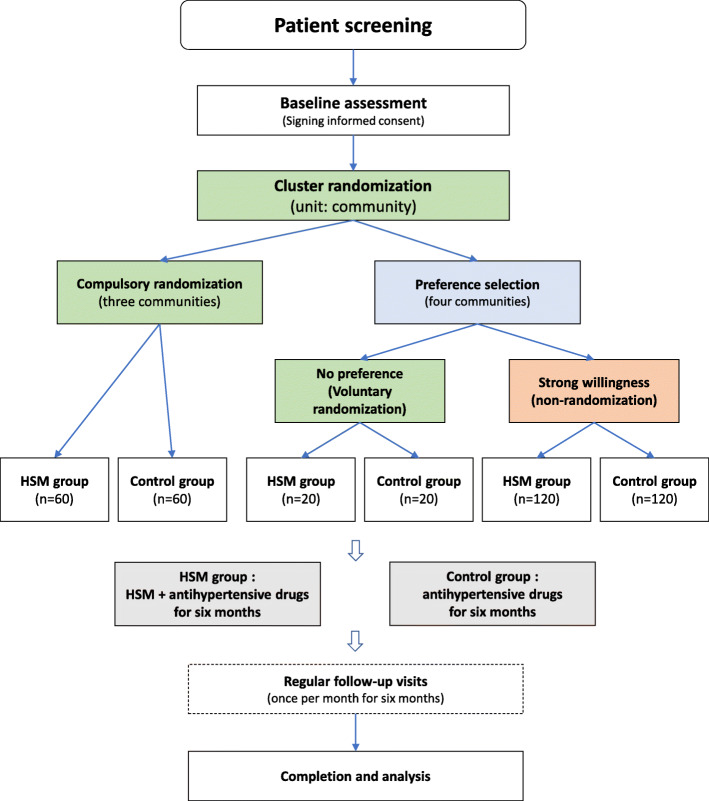
Flowchart of the study. Abbreviation: HSM, heat-sensitive moxibustion

This study will be a multicenter, pragmatic, parallel-group, superiority RCT with patient-preference arms. We will recruit patients from seven communities (Wuliangdian, Nangang, Jinsheng, Shajing, Hongmiao, Jinghexinggong, and Erliuling) to which the Affiliated Hospital of Jiangxi University of Chinese Medicine provides primary healthcare services. These communities will first be randomized into a compulsory randomization cluster and a patient preference cluster. For the former, a completely random grouping scheme to the moxibustion group (HSM + their original antihypertensive regimen) or the control group (their original antihypertensive regimen alone) will be used for individual patients; in the latter, patients can directly enter the moxibustion or control group based on their preference for managing hypertension, whereas the patients will be randomized if they have no clear preference. This grouping method will result in four randomized arms and two nonrandomized arms (Fig. 1).

### Study population

Patients who meet the following inclusion criteria will be eligible for the study: (1) diagnosis of primary hypertension for at least 6 months according to the criteria in the International Society of Hypertension’s 2020 Clinical Practice Guideline for Hypertension [17]; i.e., SBP ≥ 140 mmHg and/or DBP ≥ 90 mmHg as office-based or clinical repeated measurements without antihypertensive medication [17]; (2) age between 18 and 80 years; (3) the use of antihypertensive drugs limited to calcium channel blockers and/or angiotensin II receptor blockers; and (4) no acupoint stimulation therapy (e.g., acupuncture, moxibustion, acupressure, etc.) for treating hypertension within the last month. In addition, patients in the HSM group must be able to stimulate at least one heat-sensitized sensation around any main acupoint.

Patients with the following conditions will not be eligible for participation: (1) SBP ≥ 180 mmHg or DBP ≥ 110 mmHg despite the use of antihypertensive drugs; (2) secondary hypertension caused by acute glomerulonephritis, renal artery stenosis, primary aldosteronism, pheochromocytoma, aortic stenosis, etc.; (3) pregnancy or lactation; (4) allergy to moxa smoke or moxa leaves/velvet; and (5) complications of any disease not compatible with HSM according to the Technical Practice Guideline of Heat-Sensitive Moxibustion [18], such as acute cerebral hemorrhage, hypertensive crisis, sensory impairment, and severe mental illness.

### Allocation

For randomization, the “blockrand” function in R 4.1.0 (Ross Ihaka, Robert Gentlemen, New Zealand) will be employed to generate random sequences. We will first divide the seven communities into the compulsory randomization cluster (three communities) and patient preference cluster (four communities) via cluster randomization. Then, we will generate a random sequence with a block size of four to randomly assign the patients into the HSM or control group in a 1:1 ratio in a compulsory or voluntary manner.

The random sequence will be concealed using scratch cards printed with a random number corresponding to the participation order. Until the coating is scratched, neither the patients nor the investigators will have knowledge about the group assignments. An independent investigator who is not involved in the implementation and follow-up of this study will generate and save the randomization sequence and prepare scratch cards. Although blinding is impossible for the patients and physicians, the data analysts will not be aware of the treatment allocation.

Patients who have a clear preference may be free to choose to enter the HSM or control group, with an allocation ratio of 1:1. Hence, when either nonrandomized group is full, the remaining patients will have to enter another nonrandomized group.

### Interventions and cointerventions

In the HSM group, patients will self-administer HSM to treat hypertension. At trial entry, physicians will follow a predetermined standard operating procedure to stimulate the patient for heat-sensitized sensations using round-trip meridian, circling, and sparrow-pecking techniques around any of the acupoints at Yongquan (KI1), Shenque (CV8), and Baihui (GV20) as well as Dazhui (GV14), Quchi (LI11), Hegu (LI4), Taichong (LR3), and Zusanli (ST36). The former three are the main acupoints, and the latter four are the matching acupoints. The patients will select one main acupoint and one matching acupoint with the strongest heat-sensitized sensation as acupoints for HSM self-administration. For bilateral matching acupoints (i.e., Quchi, Zusanli, Hegu, and Taichong), the side with the stronger heat-sensitized sensation will be selected. The administration of HSM at the matching acupoint can be abandoned if all fail to stimulate heat-sensitized sensations.

After acupoint selection, the physicians will explain in detail the technique of HSM to the patients or their families, including how to use the moxibustion jar, ignition, and safety precautions (e.g., preventing burns and smoke evacuation). When dose saturation is achieved (i.e., the heat-sensitized sensations disappear), HSM can be stopped, or the patient can wait until the moxa column has burned completely (approximately 30 min). We will recommend that patients administer HSM once per day at home or at a community healthcare center. Driven by the pragmatic design, we will allow for a lower frequency of HSM when patients are busy, but the procedure must be completed at least two times per week.

Patients in both the HSM and control groups will maintain the use of their original antihypertensive regimen, including antihypertensive drugs or no treatment. The study treatments will last 6 months for all patients. During the trial period, all patients will be prohibited from receiving other acupoint stimulation therapies, such as acupuncture, acupressure, transcutaneous electrical stimulation, or acupoint application therapy. Regular treatment for comorbidities, such as glucose-lowering drugs for patients with diabetes and lipid-lowering drugs for those with dyslipidemia, will be allowed.

### Outcomes

#### Primary outcome

The primary outcome is a change in mean SBP (mmHg) at month 6, which will be measured using a uniform Omron upper-arm electronic blood pressure monitor at each study site. The measurement will be performed when the patient is in a resting state.

#### Secondary outcomes

1. A change in mean DBP (mmHg) at month 6 as measured with SBP.
2. The response to treatment at month 6, which is defined as an SBP reduction of ≥ 20 mmHg or DBP reduction of ≥ 10 mmHg for all patients, achieving SBP < 140 mmHg and DBP < 90 mmHg for patients ≥ 65 years or SBP < 130 mmHg and DBP < 80 mmHg for patients < 65 years [17].
3. A change in the mean dosage of the original antihypertensive drug at month 6. At each visit, the physician will decide whether to adjust the original antihypertensive regimen administered to control blood pressure within the ideal range according to the blood pressure measurement, which will be recorded to assess this outcome.
4. A change in mean QoL at month 6, as assessed by the Euro-QoL-5 Dimensions-5 Levels (EQ-5D-5L) questionnaire [19]. This questionnaire includes five items that evaluate QoL in terms of mobility, self-care, usual activities, pain/discomfort, and anxiety/depression. Each item receives a score from 1 to 5 (1, optimal QoL; 5, extremely poor QoL), and a comprehensive index value is calculated from these items, ranging from 0 (extremely poor QoL) to 1 (optimal QoL). The questionnaire also has an overall health status item assessed by a visual analog scale and scored between 0 and 100. A higher score indicates a better overall health status.
5. A change in the mean severity of hypertensive symptoms scored by the symptom scale in the Guiding Principles of Clinical Research on New Drugs of Chinese Medicines at month 6 [20]. The scale assesses 24 hypertension-related symptoms. Each item receives a score of 0 to 3, and a higher score indicates higher severity of that symptom; the total score is the sum of the individual item scores and ranges from 0 to 72 (see details in Additional file 1).
6. Incidence of new-onset cardiovascular events at month 6, including angina, myocardial infarction, and stroke. These data will be collected from patients and family self-reports.
7. Patient compliance with HSM at each visit. This will be determined by the dosage of each administration and the weekly frequency of applications.
8. Cost-effectiveness at month 6, as calculated by comparing the ratio of total treatment cost (China Yuan) to the rate of patients who achieve the targeted response to treatment between the HSM and control groups. Total and antihypertensive treatment costs will be analyzed. Outpatient and inpatient treatment frequencies and costs will also be compared.

#### Safety outcomes

The incidence of any adverse event (AE) and HSM-related AEs will be recorded at each visit. In this trial, an AE is defined as any adverse symptom or occurrence not related to the natural progression of the patient’s original disease. We will record the time, location, course, clinical manifestations, management, and outcome for each AE. The causal relationship between HSM and AEs will be assessed based on five aspects: HSM time and location correlations; whether the AE can be explained by other interventions; reports in previous literature and a plausible biological mechanism; remission or disappearance after HSM discontinuation; and recurrence after HSM resumption [21]. Severe AEs are defined as AEs that are fatal, life-threatening or disabling, or require prolonged hospitalization; these AEs will be immediately reported to the Ethics Committee and the provincial health administration.

### Visit schedule

**Fig. 2:**
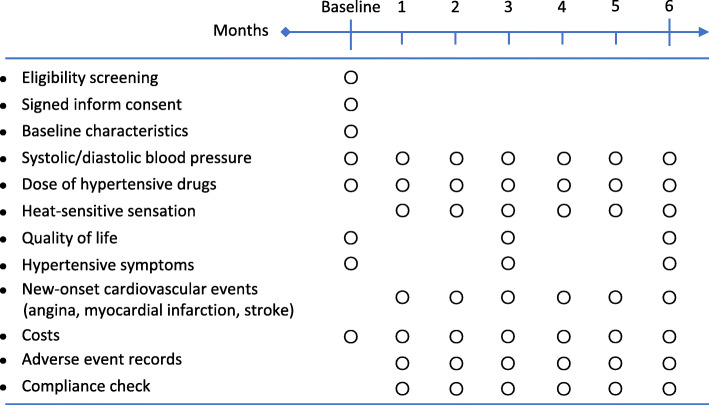
Schedule of study visits

Patients will attend a baseline visit and 6 monthly follow-up visits. The follow-up visits will be scheduled every 30 ± 3 days. If a patient does not complete a follow-up visit within the scheduled period for any reason, we will not collect follow-up data for that time period, and the data for that visit will be considered missing. Patients who fail to attend three consecutive follow-up visits within the scheduled time frame will be excluded from the study. At the baseline visit, we will collect information about demographics, comorbidities, medication history, anthropometric measurements (blood pressure, heart rate, height, and weight), and QoL and hypertensive symptom scales. At each follow-up visit, we will record blood pressure, heart rate, new-onset disease or disease progression, treatment and expenses for the month, and AE data. As a basis for patient compliance judgment and subgroup analysis, we will also record the details of HSM, including the actual dosage, frequency, acupoints, and heat-sensitized sensations at each follow-up visit. QoL and hypertensive symptoms will be assessed at the 3- and 6-month follow-ups. The detailed visit schedule is presented in Fig. 2.

### Data collection and management

At each study site (i.e., community healthcare center), a community physician will be responsible for patient recruitment, HSM training, and outcome assessment; a clinical coordinator will be responsible for regular contact with patients with regard to follow-up visits, recording basic information, and distributing moxibustion materials. Prior to the start of the trial, all investigators will be trained on the standard operating procedure and complete simulated baseline and follow-up visits before being allowed to conduct the investigation.

Raw data will be recorded on paper-based case report forms stored in a lockbox at each study site. Electronic data entry will be performed at any interim analysis and at the end of follow-up by an approach in which two clinical coordinators independently and repeatedly enter the data into standardized forms created in Microsoft Access (Microsoft Corporation, USA). The electronic data set will be anonymized and kept confidential. Only the blinded data analyst can access the data set.

The following trial committees will be established for quality assurance: a steering committee consisting of acupuncturists, cardiologists, methodologists, and statisticians and responsible for study design, maintenance of the quality of study conduct, safety monitoring, and study publication; a coordinating center consisting of the principal coordinating investigator and the coordinating investigators of each community and responsible for the overall study conduct, interactions with the communities and sponsors, and reporting trial advancements to the steering committee; an independent endpoint adjudication committee consisting of cardiologists and acupuncturists and responsible for reviewing the assessment of the primary endpoint and subjective outcomes; and an independent data-monitoring committee consisting of acupuncturists, cardiologists, methodologists, and statisticians for monitoring the data management, directing the interim analysis, and deciding whether to continue the trial based on the results of safety, efficacy, and compliance assessment.

### Recruitment and compliance enhancement

Patients will be invited to participate in the study by their primary care physician at each site. The recruitment advertisement will also be disseminated through posters and the internet. A full understanding of the 6-month follow-up schedule and signing of the informed consent form are prerequisites for participation. Patients’ long-term trust in their physicians is an essential guarantee of their willingness to participate in the study and comply with the protocol. At each visit, patients will obtain a comprehensive health assessment and recommendations for adjusting their medications for hypertension and other diseases. We will also provide a number of rooms with air conditioning and fume extractors at each study site for the patients to administer HSM.

To promote recruitment and compliance, we will provide the patients in both the randomized and nonrandomized HSM groups with two boxes of moxa rolls (108 rolls) per month, which is enough for consumption during the month. At the baseline visit, we will also give the patients two free moxibustion jars, each equipped with a fleece bag that can be slipped over the outside to prevent burns. At the follow-up visits, we will ask the patients to bring the moxibustion jars with them to determine whether the reported frequency of HSM matches the thickness of soot accumulation on the jars. If a moxibustion jar breaks, we will replace it with a new jar. Patients randomized into the control group will receive two more boxes of moxa rolls and two more moxibustion jars than those in the HSM group after completing the study. Because they are not interested in performing HSM, after completing the study, the patients who voluntarily enter the control group will be given a blood pressure monitor that is of equal value to the moxibustion materials.

### Sample size estimation

The primary purpose of sample size estimation is to meet the need for statistical power for primary outcome comparison between randomized groups. Based on the results of our previous nonrandomized studies (not yet published) and expert opinion, we assume that the SBP in the HSM group will be on average 5 mmHg lower than that in the control group after 6 months of HSM treatment, with a common standard deviation of 8 mmHg between the groups. With a type I error probability “α” of 0.05 and type II error probability “β” of 0.20 and the allowance of a 20% attrition rate, a minimum of 51 patients in either the randomized HSM or control group is needed, as calculated by the following formula for the superiority design [22]:

$$ N=2\times {\left(\frac{Z_{1-\frac{\alpha }{2}}+{Z}_{1-\beta }}{\upmu_1-{\upmu}_2}\right)}^2\times {s}^2 $$

We ultimately decided to enlarge the total sample size to 400, as a sample of this size can be supported by funding. We administered a small preference survey to 100 patients at the study sites, which showed that the ratio of patients with a preference (a strong willingness to enter the HSM or control group) to those without a preference (accept the randomization) was approximately 6:1. Considering the feasibility of the study and the need for confounder adjustment, we established a sample size for the voluntary randomization group of 40 (20 per group) such that the size of the preference selection (nonrandomized) group would be 240 (120 per group). The remaining 120 patients will be entered into the compulsory randomization group (60 per group). Figure 1 illustrates the sample size scheme for each arm.

### Statistical analysis

Data analysis of the primary outcome will be based on the full analysis set constructed by the intention-to-treat principle, whereby patients will remain in their original group regardless of contamination. The per-protocol set will also be analyzed by sensitivity analysis for the primary outcome to evaluate whether contamination impacts the direction of the effect. The secondary outcomes will also be compared based on the per-protocol set. The need to impute missing values will be determined by the proportion of and reasons for missing data. If imputation is necessary, appropriate methods, such as the last observation carried forward, “best-worst-case” scenario and multiple imputation regression modeling, will be chosen according to the distribution of the missing values.

Data analysis will be conducted separately for the randomized and nonrandomized groups, and consistency of effect directions between the two sets of analyses will be evaluated. For the randomized groups, repeated-measures analysis of variance will be employed for the continuous variables measured each month; QoL and symptom scale scores will be compared by *t* tests, and categorical variables will be compared by chi-square tests. These analyses will not be adjusted for covariates unless there are factors that are significantly unbalanced at baseline. For nonrandomized groups, we will utilize repeated-measures ANOVA, linear mixed models, and logistic regression models depending on the data distribution; potential confounders will be adjusted for, including age, body mass index (BMI), baseline SBP, duration of hypertension, type of antihypertensive regimen, diabetes, dyslipidemia, smoking, and alcohol consumption. The between-group difference will be measured by the mean differences for continuous outcomes (i.e., changes in SBP, DBP, dosage of antihypertensive drug, QoL, and hypertensive symptom scores) and the risk ratios (RRs) for binary outcomes (i.e., the response to treatment and incidence of new-onset cardiovascular events).

We are also interested in whether there are different blood pressure lowering effects between HSM regimens with different parameters, which will be explored by the following subgroup analyses with prespecified effect directions based on our clinical experience:

1. A high degree of heat-sensitized sensation is expected to be associated with better efficacy than a low degree. A scale developed in-house will be used to measure the degree of heat-sensitized sensation. The scale includes four dimensions (depth/range/distance [not available for three systemic reactions], intensity, frequency, and comfort) for 12 sensations (Additional file 2). Each dimension can receive a score of 0 to 5 points. A patient whose total score in the four dimensions for any sensation is ≥ 60% (i.e., 15 points) will be considered to have achieved a high degree of heat-sensitized sensation. This scale was tested in a sample of 64 patients and showed good reliability and validity—the Cronbach alpha coefficient was 0.892, and the factor loadings for all items were > 0.6.
2. HSM ≥ 4 times per week is expected to be associated with better efficacy than HSM < 4 times per week.
3. Heat-sensitized sensations at both the main and matching acupoints are expected to be associated with better efficacy than heat-sensitized sensations at only the main acupoint.
4. The development of nonthermal sensation (the last 7 moxibustion sensations on the scale) is expected to be associated with better efficacy than the development of only thermal sensation.

In addition, subgroup analyses comparing patients who underwent compulsory and voluntary randomization will be carried out to validate the impact of patient preference on the effects.

If the HSM group shows a better response to treatment than the control group, we will perform an economic evaluation in which the cost-effectiveness ratio and incremental cost-effectiveness ratio will be used as indicators. If a better response to treatment is associated with the frequency of HSM, the balance between the costs of moxibustion materials and outcomes will be explored. A cost-effectiveness acceptability curve will be applied to visualize information about uncertainty in the cost-effectiveness analysis.

### Dissemination policy

We will do effort to disseminate the study findings through publications in peer-reviewed journals and conference presentations.

## Discussion

Blinding is usually not feasible in RCTs involving healthcare techniques that require manual manipulation [23], including HSM. Consequently, patients will clearly know whether they are receiving the experimental intervention, and patient preference may cause uncertain effects on the study process and results [24, 25]. To reduce patient preference effects, we chose to perform a randomized patient preference trial, from which several advantages can be derived. First, this design allows us to analyze patient-preference effects by comparing the results of compulsory and voluntary randomization. Another benefit of a compulsory randomization group is avoiding being unable to achieve randomization when the proportion of patients who choose voluntary randomization is too small. Second, evaluation of randomized and nonrandomized samples will improve the reliability of the results if they can be corroborated. The evidence from nonrandom samples will also have better external validity. Third, this design will allow the study to recruit more participants, and the patients in the patient preference cluster will be more adherent to the protocol and less likely to be lost to follow-up.

A potential limitation of this study is that the patients in the HSM group may not easily adhere to the 6-month HSM treatment regimen, especially when the weather is hot. Although we will provide air-conditioned rooms at each study site for them to administer HSM, there is no guarantee that all patients will be willing to visit during the hot months of the summer. Nevertheless, patient compliance is an important outcome, and poor compliance would suggest that this community-based HSM model is not feasible. In addition, although the hypertensive symptom scale implemented in this study is from an authoritative guideline and has been used in a number of studies [15] to date, there is no direct evidence to support its reliability and validity.

In summary, this will be the first RCT to evaluate the efficacy, safety, economics, and compliance profile of community-based HSM for primary hypertension and will produce nonrandomized evidence. The study will have a sufficient sample size to obtain accurate estimates, as well as sufficient 6-month follow-up data to verify long-term safety and patient compliance. If proven to be a safe and cost-effective method, HSM is expected to be widely promoted in primary healthcare for the treatment of primary hypertension in low- and middle-income countries. Such efforts may also promote the development of relevant industries, such as mugwort cultivation and moxibustion material production.

## Trial status

The protocol version is 1.1, developed on March 1, 2021, and updated on January 17, 2022. The trial began recruiting on 10 March 2021 and is anticipated to finish in June 2022.

## Supplementary Information

**Additional file 1.** Hypertensive symptoms scale

**Additional file 2.** Heat-sensitized sensation self-evaluation scale

**Additional file 3.** Informed consent form (translated)

## Data Availability

The datasets collected during the current study are available from the corresponding author on reasonable request.

## Abbreviations

AE: Adverse event
BMI: Body mass index
DBP: Diastolic blood pressure
EQ-5D-5L: Euro-QoL-5 Dimensions-5 Levels questionnaire
HSM: Heat-sensitive moxibustion
QoL: Quality of life
RCT: Randomized controlled trial
SBP: Systolic blood pressure
