# Supplementary material for: Community-based heat-sensitive moxibustion for primary hypertension: study protocol for a randomized controlled trial with patient-preference arms
Source: Trials. 2022 Feb 16;23:154. doi: 10.1186/s13063-022-06092-4 (PMC8848640; doi:10.1186/s13063-022-06092-4)
Supplement: Supplementary file 1 — Additional file 1. Hypertensive symptoms scale [file 13063_2022_6092_MOESM1_ESM.pdf]

Additional file 1: Hypertensive Symptoms Scale

Symptoms	Normal	Mild	Moderate	Severe
**Dizziness**	☐None	☐Slight and Sporadic	☐Unable to walk	☐Unable to stand
**Headache**	☐None	☐Slight and Sporadic	☐Persistent but tolerable	☐Unbearable
**Irritability**	☐None	☐0ccasionally upset	☐Prone to irritability	☐Uncontrolled irritability
**Soreness of waist**	☐None	☐Mild and can be relieved	☐Persistent and aggravated by work	☐Cannot be alleviated after rest
**Knee weakness**	☐None	☐Slight weakness	☐Cannot bear heavy loads	☐Unable to walk
**Feverish sensation with dysphoria in chest, palms and soles**	☐None	☐Feverish sensation in palms and soles at night	☐Feverish sensation and dysphoria in chest, palms and soles	☐Unwilling to get dress and cover up
**Heavy-headedness**	☐None	☐Slightly feeling heavy in the head	☐Heavy-headedness as covering a cloth	☐Head tight like wearing a hat
**Chest distress**	☐None	☐Slight chest distress	☐Obvious chest distress with sign-like breathing	☐Severe chest distress like suffocation
**Expectoration**	☐None	☐Sporadically clear sputum	☐Retching with sticky sputum	☐Vomiting with a lot of sputum
**Chills**	☐None	☐Slight	☐Obvious chills and cold limbs	☐Chills and cold limbs to wear more clothes and blankets
**Flushed face**	☐None	☐Slightly flushed	☐Visible redness	☐Blushing as if wearing make-up
**Bloodshot eyes**	☐None	☐Slight redness	☐Evident redness	☐Like the eyes of a turtledove
**Xerostomia**	☐None	☐Slight	☐Dry mouth and reduced saliva	☐Dry mouth requiring frequent drinking
**Bitterness in the mouth**	☐None	☐Slight bitterness in the morning	☐Bitter mouth resulting in lack of taste	☐Bitter and astringent in mouth
**Constipation**	☐None	☐Dry stools, once a day	☐Constipated stool, once every two days	☐Difficult stools, once for several days
**Dark urine**	☐None	☐Slightly yellow urine	☐Yellow and scanty urine	☐Dark urine with difficulty in urinating
**Palpitation**	☐None	☐Occasional mild palpitations	☐Frequent palpitations	☐Prolonged and severe palpitation
**Insomnia**	☐None	☐Slight reduction in sleep duration	☐Sometimes insomnia	☐Prolonged difficulty sleeping
**Tinnitus**	☐None	☐Slightly and occasional tinnitus	☐Frequent tinnitus and hearing loss	☐Persistent tinnitus and severe hearing loss
**Amnesia**	☐None	☐Occasional forgetfulness but can still be remembered	☐Frequently and not easy to remember	☐Cannot recall
**Tastelessness**	☐None	☐Slight loss of taste sensation	☐Obvious loss of taste sensation	☐Loss of appetite due to lack of taste sensation
**Inappetence**	☐None	☐Slightly reduction in diet	☐Reduction in diet	☐Significant reduction in diet
**Dyspnea**	☐None	☐Shortness of breath after activity	☐Shortness of breath without activity	☐Severe shortness of breath
**Frequent nocturia**	☐None	☐One time	☐Two or three times	☐More than three times
