# Supplementary material for: Community-based heat-sensitive moxibustion for primary hypertension: study protocol for a randomized controlled trial with patient-preference arms
Source: Trials. 2022 Feb 16;23:154. doi: 10.1186/s13063-022-06092-4 (PMC8848640; doi:10.1186/s13063-022-06092-4)
Supplement: Supplementary file 2 — Additional file 2. Heat-sensitized sensation self-evaluation scale [file 13063_2022_6092_MOESM2_ESM.pdf]

Additional file 2. Heat-sensitized Sensation Self-evaluation Scale

Sensation Types	Dimensions	Options for each dimension
1 . Do you feel heat penetrating from the surface of the skin to the inside? □$_0$ No □$_1$ Yes➔	Comfort	□$_1$ None □$_2$ Slight □$_3$ Moderate □$_4$ Considerable □$_5$Extreme
	Depth	□$_1$ Very shallow □$_2$ shallow □$_3$ Moderate □$_4$ Deep □$_5$ Very deep
	Intensity	□$_1$ Very weak □$_2$ Weak □$_3$ Moderate □$_4$ Strong □$_5$ Very strong
	Frequency (per 10 times)	□$_1$ ≤1 time □$_2$ 2-4 times □$_3$ 4-6 times □$_4$ 7-9 times □$_5$ every time
2. Do you feel heat spreading from the place of moxibustion? □$_0$ No □$_1$ Yes ➔	Comfort	□$_1$ None □$_2$ Slight □$_3$ Moderate □$_4$ Considerable □$_5$Extreme
	Range	□$_1$ Very small □$_2$ Small □$_3$ Moderate □$_4$ Large □$_5$ Very large
	Intensity	□$_1$ Very weak □$_2$ Weak □$_3$ Moderate □$_4$ Strong □$_5$ Very strong
	Frequency (per 10 times)	□$_1$ ≤1 time □$_2$ 2-4 times □$_3$ 4-6 times □$_4$ 7-9 times □$_5$ every time
3. Do you feel heat transfer from one direction to a distant place? □$_0$ No □$_1$ Yes➔	Comfort	□$_1$ None □$_2$ Slight □$_3$ Moderate □$_4$ Considerable □$_5$Extreme
	Distance	□$_1$ Very close □$_2$ Close □$_3$ Moderate □$_4$ Far □$_5$ Very far
	Intensity	□$_1$ Very weak □$_2$ Weak □$_3$ Moderate □$_4$ Strong □$_5$ Very strong
	Frequency (per 10 times)	□$_1$ ≤1 time □$_2$ 2-4 times □$_3$ 4-6 times □$_4$ 7-9 times □$_5$ every time
4. Do you feel that the place of moxibustion are not or not so hot, but hot in a distant place? □$_0$ No □$_1$ Yes➔	Comfort	□$_1$ None □$_2$ Slight □$_3$ Moderate □$_4$ Considerable □$_5$Extreme
	Distance	□$_1$ Very close □$_2$ Close □$_3$ Moderate □$_4$ Far □$_5$ Very far
	Intensity	□$_1$ Very weak □$_2$ Weak □$_3$ Moderate □$_4$ Strong □$_5$ Very strong
	Frequency (per 10 times)	□$_1$ ≤1 time □$_2$ 2-4 times □$_3$ 4-6 times □$_4$ 7-9 times □$_5$ every time
5 . Do you feel that the surface of the skin is not or not so hot but hot in the depths? □$_0$ No □$_1$ Yes➔	Comfort	□$_1$ None □$_2$ Slight □$_3$ Moderate □$_4$ Considerable □$_5$Extreme
	Depth	□$_1$ Very shallow □$_2$ shallow □$_3$ Moderate □$_4$ Deep □$_5$ Very deep
	Intensity	□$_1$ Very weak □$_2$ Weak □$_3$ Moderate □$_4$ Strong □$_5$ Very strong
	Frequency (per 10 times)	□$_1$ ≤1 time □$_2$ 2-4 times □$_3$ 4-6 times □$_4$ 7-9 times □$_5$ every time
6. Do you have a feeling like ants crawling at the place of moxibustion? □$_0$ No □$_1$ Yes➔	Comfort	□$_1$ None □$_2$ Slight □$_3$ Moderate □$_4$ Considerable □$_5$Extreme
	Range	□$_1$ Very small □$_2$ Relatively small □$_3$ Moderate □$_4$ larger □$_5$ Very large
	Intensity	□$_1$ Very weak □$_2$ Weak □$_3$ Moderate □$_4$ Strong □$_5$ Very strong
	Frequency (per 10 times)	□$_1$ ≤1 time □$_2$ 2-4 times □$_3$ 4-6 times □$_4$ 7-9 times □$_5$ every time
7 . Do you feel a swelling sensation at the place of moxibustion? □$_0$ No □$_1$ Yes➔	Comfort	□$_1$ None □$_2$ Slight □$_3$ Moderate □$_4$ Considerable □$_5$Extreme
	Range	□$_1$ Very small □$_2$ Relatively small □$_3$ Moderate □$_4$ larger □$_5$ Very large
	Intensity	□$_1$ Very weak □$_2$ Weak □$_3$ Moderate □$_4$ Strong □$_5$ Very strong
	Frequency (per 10 times)	□$_1$ ≤1 time □$_2$ 2-4 times □$_3$ 4-6 times □$_4$ 7-9 times □$_5$ every time
8. Do you feel soreness at the place of moxibustion? □$_0$ No □$_1$ Yes➔	Comfort	□$_1$ None □$_2$ Slight □$_3$ Moderate □$_4$ Considerable □$_5$Extreme
	Range	□$_1$ Very small □$_2$ Relatively small □$_3$ Moderate □$_4$ larger □$_5$ Very large
	Intensity	□$_1$ Very weak □$_2$ Weak □$_3$ Moderate □$_4$ Strong □$_5$ Very strong
	Frequency (per	□$_1$ ≤1 time □$_2$ 2-4 times □$_3$ 4-6 times □$_4$ 7-9 times □$_5$ every time

	10 times)	
**9. Do you have a feeling like cool air coming out at the place of moxibustion?** ☐$_0$ No ☐$_1$ Yes➔	Comfort	☐$_1$ None ☐$_2$ Slight ☐$_3$ Moderate ☐$_4$ Considerable ☐$_5$Extreme
	Range	☐$_1$ Very small ☐$_2$ Relatively small ☐$_3$ Moderate ☐$_4$ larger ☐$_5$ Very large
	Intensity	☐$_1$ Very weak ☐$_2$ Weak ☐$_3$ Moderate ☐$_4$ Strong ☐$_5$ Very strong
	Frequency (per 10 times)	☐$_1$ ≤1 time ☐$_2$ 2-4 times ☐$_3$ 4-6 times ☐$_4$ 7-9 times ☐$_5$ every time
**10. Do you feel that your stomach and intestines are moving faster?** ☐$_0$ No ☐$_1$ Yes➔	Comfort	☐$_1$ None ☐$_2$ Slight ☐$_3$ Moderate ☐$_4$ Considerable ☐$_5$Extreme
	Intensity	☐$_1$ Very weak ☐$_2$ Weak ☐$_3$ Moderate ☐$_4$ Strong ☐$_5$ Very strong
	Frequency (per 10 times)	☐$_1$ ≤1 time ☐$_2$ 2-4 times ☐$_3$ 4-6 times ☐$_4$ 7-9 times ☐$_5$ every time
**11. Do you feel face flushing or forehead sweating but actually no moxibustion on these places?** ☐$_0$ No ☐$_1$ Yes➔	Comfort	☐$_1$ None ☐$_2$ Slight ☐$_3$ Moderate ☐$_4$ Considerable ☐$_5$Extreme
	Intensity	☐$_1$ Very weak ☐$_2$ Weak ☐$_3$ Moderate ☐$_4$ Strong ☐$_5$ Very strong
	Frequency (per 10 times)	☐$_1$ ≤1 time ☐$_2$ 2-4 times ☐$_3$ 4-6 times ☐$_4$ 7-9 times ☐$_5$ every time
**12. Do you feel heat in the palms and soles but actually no moxibustion on these places?** ☐$_0$ No ☐$_1$ Yes➔	Comfort	☐$_1$ None ☐$_2$ Slight ☐$_3$ Moderate ☐$_4$ Considerable ☐$_5$Extreme
	Intensity	☐$_1$ Very weak ☐$_2$ Weak ☐$_3$ Moderate ☐$_4$ Strong ☐$_5$ Very strong
	Frequency (per 10 times)	☐$_1$ ≤1 time ☐$_2$ 2-4 times ☐$_3$ 4-6 times ☐$_4$ 7-9 times ☐$_5$ every time
