# Supplementary material for: Community-based heat-sensitive moxibustion for primary hypertension: study protocol for a randomized controlled trial with patient-preference arms
Source: Trials. 2022 Feb 16;23:154. doi: 10.1186/s13063-022-06092-4 (PMC8848640; doi:10.1186/s13063-022-06092-4)
Supplement: Supplementary file 3 — Additional file 3. Informed consent form (translated) [file 13063_2022_6092_MOESM3_ESM.pdf]

Additional file 3. Informed consent form (translated)

Community-based heat-sensitive moxibustion self-management for primary hypertension: a multi-center pragmatic randomized patient-preference controlled trial

Patient Informed Consent Form

Dear patients, you are being invited to participate in a research project sponsored and hosted by the research team of Jiangxi University of Chinese Medicine and the Affiliated Hospital of Jiangxi University of Chinese Medicine, entitled "Community-based heat-sensitive moxibustion self-management for primary hypertension: a multi-center pragmatic randomized patient-preference controlled trial". Here we inform to you the details of this trial.

1. Background and objectives

Hypertension is a high-risk factor for the development of cardiovascular diseases, most of which are asymptomatic or manifest only as mild headache and dizziness at the initial stage, but the long-term hypertensive effect can lead to a series of cardiovascular diseases, such as atherosclerosis formation, stroke, and even life-threatening complications in severe cases. The objectives of this study are to evaluate the efficacy and safety of community-based heat-sensitive moxibustion self-management in primary hypertension.

2. Introduction to heat-sensitive moxibustion

Heat-sensitive moxibustion is a new moxibustion therapy that uses the moxa heat generated by the ignited moxa material at acupoint with heat-sensitive status to stimulate heat penetrating, heat diffusion, heat conduction, local not (micro) heat but distal heat, surface not (micro) heat but deep heat, and non-thermal sensation. Applying individualized dose of saturated desensitizing moxibustion on heat-sensitive acupoints

can improve the efficacy of moxibustion. Heat-sensitive moxibustion is an original clinical technique of Professor Rixin Chen's team at Jiangxi University of Chinese Medicine. Heat-sensitive moxibustion can stimulate the meridian qi to reach the place where the qi reaches the disease, so that greatly improve the efficacy of traditional suspension moxibustion, which is now popularized and used in the whole China and even the world.

3. Conditions to be met for participation in this study

The research team members in charge of the study will discuss with you the requirements for participation in the study. You will be asked to give a complete description of your blood pressure and disease history to the study team members. You may enter the screening period of this study if you meet the following criteria and, in the judgment of the research team member, you may participate in this study if you meet all the following criteria.

(1) Inclusion criteria

1) Diagnosed with essential hypertension, with a course more than 6 months.
2) Ages: 18-80 years.
3) Did not receive acupoint stimulation therapies for hypertension in the last month.
4) The original antihypertensive drugs are calcium channel blockers and/or angiotensin II receptor blockers.
5) Patients in the heat-sensitive moxibustion groups need to develop at least one type of moxibustion sensation around the following acupoints: Yongquan, Baihui, Shenque, Quchi, Zusanli, Hegu, Taichong and Dazhui.
6) Consent to sign an informed consent form

(2) Exclusion criteria

1) Systolic blood pressure ≥ 180mmHg and/or diastolic blood pressure ≥ 110mmHg after taking antihypertensive drugs;
2) Secondary hypertension.
3) Pregnancy and lactation;
4) Allergic to moxibustion devices, moxa smoke or Artemisia argyi.

5) Complicated with severe diseases that are not recommended for heat-sensitive moxibustion, such as acute cerebral hemorrhage, hypertensive crisis, sensory disturbances, serious mental diseases, etc.

4. Research process

The entire study period will be 6 months, with 7 visits after enrollment, including the visit at baseline and one visit per month thereafter. We will collect the following information: general conditions (gender, age, physical examination, lifestyle habits, and education), diagnostic/treatment records (comorbidities, medication use, medical costs, outpatient and inpatient records, and application of heat-sensitive moxibustion), records of treatment outcomes (blood pressure measurements, quality of life, hypertensive symptoms, hypertensive complications, heat-sensitive sensation, etc.), and safety.

5. Grouping method

We will randomly assign you to a preference selection cluster or a compulsory randomization cluster. If you enter the preference selection cluster, you may decide whether to enter the moxibustion or control group, or to receive randomization. If you enter the compulsory randomization cluster, you will be randomly assigned to the moxibustion or control group.

(1) If you enter the moxibustion group, you will be required to self-administer heat-sensitive moxibustion treatment based on the original type and dose of your antihypertensive medication. A professional heat-sensitive moxibustion doctor will explore and determine the heat-sensitive acupoints for you before the trial begins, then you or your family will be trained in the self-management method of heat-sensitive moxibustion. After mastering the implementation of heat-sensitive moxibustion, you may self-administer moxibustion in your home or the moxibustion place in the community health service center. The dose of moxibustion is until the heat-sensitive sensation disappears, and the frequency is recommended once a day (you can adjust the frequency as needed, but at least twice a week). The course of treatment is six months.

(2) If you enter the control group： you will be required to self-administer heat-sensitive moxibustion treatment based on the original type and dose of your antihypertensive medication. There are no other interventions.

(3) Other treatment：Regular treatment for comorbidities will be no limitations. During the trial period, both groups will be prohibited from receiving other acupoint stimulation therapies, such as acupuncture, acupressure, or acupoint application therapy.

6. Possible benefits of participating in the study

Your medical condition may improve as a result of participating in this trial. Expected improvements may include lower blood pressure, reduced symptoms of hypertension, and improved quality of life, but we do not and are not able to guarantee that you will receive benefits from this study.

The cost of treatment related to heat-sensitive moxibustion for participation in this study will be provided free of charge by the sponsor (including moxa rolls and moxibustion jars) until the end of the trial. If you are randomized to the control group, you will receive the same amount of free moxibustion material as the moxibustion group at the end of the trial; if you voluntarily choose to enter the control group, you will receive an electronic blood pressure monitor of the same value as the moxibustion material at the end of the trial. In addition, you will receive a comprehensive evaluation of your disease at each follow-up visit and will receive treatment instructions from your doctor.

The cost of tests associated with participation in this study will be provided free of charge by the sponsor, including blood pressure measurement, quality of life assessment, and hypertensive symptom assessment.

7. Risk and prevention plan in the trial

The risks for patients in the heat-sensitive moxibustion group in this study is possible burns due to improper handling of moxibustion and possible allergy to moxa material or moxa smoke. The control group does not have any additional health risks.

You will be given thorough safety training prior to the start of the study to avoid burns as much as possible. If burns occur and small local blisters appear, they can be allowed to absorb naturally as long as care is taken to do not abrade them; if the blisters are large or appear septic, prompt medical attention should be sought and the research team will be responsible for further treatment and bear the cost of treatment. A small number of patients may have mild allergic reactions to moxa material or moxa smoke,

and if allergic reactions appear, please discontinue the heat-sensitive moxibustion treatment. There is no evidence that moxibustion smoke can cause other adverse effects on the body.

There are risks, discomforts and inconveniences associated with any scientific study, so you should consider carefully before agreeing to participate in this study.

8. Participation/withdrawal/termination

Participation in this study is entirely voluntary. You may decline to participate in the study or withdraw from the study at any time during the study, and this will not affect your relationship with your doctor and result in any loss of medical or other benefits to you.

9. Confidentiality measures

Any personal data about you will be anonymized and kept strictly confidential for scientific use only. Any researcher needing to use your data database will be required to sign a confidentiality agreement and use and analyze the data under strict supervision and management. Although your data may be monitored by relevant authorities (e.g., ethics committees and data and safety monitoring committees), we ensure that your personal information will not be disclosed to the public.

10. Other matters

You will not be reimbursed for the antihypertensive medication you take or for treatment and tests required for other comorbidities.

This consent form is in two copies, one for the study unit and one for the subject. If there is any violation of the study protocol during the study, you may complain directly to the Ethics Committee.

Informed Consent Form - Consent Signature Page

I have been informed of the purpose and methods of this trial and the possible risks, discomforts, and benefits.

I am certain that I have spent sufficient time reading and understanding the above and that my doctor has explained to me the medical terms used in it. My doctor has answered all questions I have asked about the study to my satisfaction.

I understand that I may voluntarily withdraw from this study at any time without jeopardizing the future doctor-patient relationship and treatment. I understand that if I have any questions during the trial, I should contact my supervising physician promptly.

I voluntarily participate in this study and agree to cooperate with the physician in accordance with the study methods and the informed consent form and to complete this study carefully.

Sign of subject or designated agent：__________________

Date: _________ Year _______ Month _______Day

I have informed the patient (or designated agent) truthfully about the purpose, content, benefits, and possible adverse effects of this study. I have asked the patient if he or she has any questions about this study and have explained them to the best of my ability.

Signature of the study investigator：__________________

Date: _________ Year _______ Month _______Day
